# Indicadores de monitoramento e avaliação dos Centros de Parto Normal Peri-hospitalares: resultados do estudo *Nascer nas Casas de Parto do Brasil*

**DOI:** 10.1590/0102-311XPT175024

**Published:** 2025-07-21

**Authors:** Cláudia de Azevedo Aguiar, Nathalie Leister, Jamile Claro de Castro Bussadori, Maria Luiza Gonzalez Riesco, Thais Trevisan Teixeira

**Affiliations:** 1 Universidade Federal do Triângulo Mineiro, Uberaba, Brasil.; 2 Universidade Federal de São Carlos, São Carlos, Brasil.; 3 Escola de Enfermagem, Universidade de São Paulo, São Paulo, Brasil.; 4 City St. George’s, University of London, London, U.K.; 5 Centro Universitário Adventista de São Paulo, São Paulo, Brasil.

**Keywords:** Centros de Assistência à Gravidez e ao Parto, Gravidez, Trabalho de Parto, Parto, Puerpério, Birthing Centers, Pregnancy, Obstetric Labor, Parturition, Postpartum Period, Centros de Asistencia al Embarazo y al Parto, Embarazo, Trabajo de Parto, Parto, Periodo Posparto

## Abstract

O objetivo deste artigo é analisar os indicadores de monitoramento e avaliação dos Centros de Parto Normal Peri-hospitalares (CPNp) do Brasil. Trata-se de um estudo transversal, aninhado à pesquisa *Nascer no Brasil II*. Fizeram parte do estudo todos os oito CPNp do país que registraram mais de 100 partos em 2019. As fontes de dados foram as entrevistas com gestoras dos serviços. Os dados se referem ao ano de 2020 ou 2021 e foram coletados utilizando-se entrevista e instrumento fechado disponibilizado on-line. As variáveis correspondem a todos os 12 indicadores de monitoramento e avaliação previstos na portaria de habilitação dos CPNp pelo Ministério da Saúde. Os resultados mostraram que, no período de um ano, foram atendidos 3.097 partos. Destes, 96,9% foram atendidos por enfermeira/obstetriz e 98,7% tiveram a presença de acompanhante de escolha da mulher. A aminiotomia foi realizada em 15,7% dos partos; a ocitocina foi utilizada em 7% e a episiotomia em 0,4% dos partos; 60,2% destes ocorreram na posição vertical; e em 32,2% o períneo permaneceu íntegro. Quanto aos recém-nascidos, 0,6% tiveram Apgar < 7 no 5º minuto e 97,6% tiveram contato pele a pele imediato com suas mães. As taxas de transferência materna intraparto, pós-parto e neonatal foram, respectivamente, 17,3%, 2,6% e 4,3%. Os indicadores dos CPNp demonstram que este modelo de atenção oferece às mães e bebês uma assistência segura, respeitosa, humano-centrada e baseada nas melhores evidências científicas.

## Abstract (en)

This study aimed to analyze the monitoring and evaluation indicators of Freestanding Birth Centers (FBC) in Brazil. This cross-sectional study was aligned with the survey *Birth in Brazil II*. All eight FBC that attended more than 100 births in 2019 were included in this study. Data referred to 2020 or 2021 and were collected from interviews with service managers and a structured instrument made available online. Variables correspond to all 12 monitoring and evaluation indicators provided for in the ordinance by the Brazilian Ministry of Health for the accreditation of FBC. Results showed that the service attended 3,097 births in a year. Nurses/midwives attended 96.9% of these births and 98.7% of them included a companion of the woman’s choice. Amniotomy was performed in 15.7% of labors; oxytocin and episiotomy were used in 7% and performed in 0.4% of them, respectively; 60.2% of births occurred in the vertical position; and the perineum remained intact in 32.2% of them. As for the newborns, 0.6% had five-minute Apgar scores < 7 and 97.6% had skin-to-skin contact with their mothers. The intrapartum, postnatal, and neonatal transfers rates totaled 17.3%, 2.6%, and 4.3%, respectively. The indicators of the FBC show that this model of care offers mothers and babies safe, respectful, human-centered care that is based on the best scientific evidence.

## Resumen

El objetivo de este estudio fue analizar los indicadores de seguimiento y evaluación de los Centros de Parto Normal Perihospitalarios (CPNp) en Brasil. Se trata de un estudio transversal, conectado a la investigación *Nacer en Brasil II*. Formaron parte del estudio las ocho CPNp del país que registraron más de 100 nacimientos en 2019. Las fuentes de datos fueron entrevistas con gerentes de servicios. Los datos se refieren al año 2020 o 2021 y se recogieron mediante entrevistas y un instrumento cerrado disponible en línea. Las variables corresponden a los 12 indicadores de seguimiento y evaluación previstos en la ordenanza que habilita el CPNp por parte del Ministerio de Salud brasileño. Los resultados mostraron que, en un periodo de un año, se atendieron 3.097 partos. De estes, el 96,9% fueron atendidos por una enfermera/partera y el 98,7% contaron con la presencia de un acompañante elegido por la mujer. Se realizó amniotomia en el 15,7% de los partos; se utilizó oxitocina en el 7% y episiotomía en el 0,4% de los partos; el 60,2% de estos ocurrieron en posición vertical; y en el 32,2%, el perineo permaneció intacto. En cuanto al recién nacido, el 0,6% tenía puntuaciones de Apgar < 7 en el quinto minuto y el 97,6% tuvieron contacto piel a piel inmediato con sus madres. Las tasas de transferencia materna intraparto, posparto y neonatal fueron, respectivamente, de 17,3%, 2,6% y 4,3%. Los indicadores del CPNp demuestran que este modelo de atención ofrece a las madres y al bebé una atención segura, respetuosa, centrada en el ser humano y basada en la mejor evidencia científica.

## Introdução

No Brasil, a atenção ao parto e ao nascimento é marcada historicamente por um modelo de assistência hospitalar centrado no médico e caracterizado por altas taxas de intervenções obstétricas, como cesarianas e partos instrumentais. Esse modelo, predominante tanto no setor público quanto no privado, tem sido alvo de críticas de especialistas em saúde materna e neonatal, além de instituições internacionais, como a Organização Mundial da Saúde (OMS), as quais recomendam a promoção de práticas mais humanizadas e baseadas em evidências para o parto normal. Nesse contexto, os Centros de Parto Normal Peri-hospitalares (CPNp) surgem como uma estratégia para a reestruturação da assistência ao parto de risco habitual no Brasil, visando, especialmente, a redução da morbimortalidade materna e neonatal ^1^.

Os CPNp são unidades de saúde específicas para o atendimento de partos de risco habitual, localizadas próximas a hospitais, de modo a garantir acesso rápido a recursos médicos e tecnológicos em caso de necessidade de transferência. Regulamentados pela *Portaria nº 11/2015* ^2^ do Ministério da Saúde, os CPNp têm como objetivo proporcionar um ambiente acolhedor e seguro, onde as gestantes podem vivenciar o parto de forma humanizada e com o mínimo de intervenções. Esses centros são conduzidos por obstetrizes e enfermeiras obstétricas, profissionais capacitados para atender partos normais de risco habitual, sem a necessidade da presença de profissionais médicos. Essa configuração é alinhada às recomendações da OMS, que enfatizam que a maior parte dos partos pode ser conduzida com segurança por estes profissionais de saúde, especialmente em cenários de risco habitual ^3^.

A criação e a expansão dos CPNp no Brasil fazem parte de uma estratégia mais ampla para reduzir a medicalização excessiva do parto e enfrentar o cenário de cesarianas em níveis alarmantes. Segundo dados do Ministério da Saúde ^4^, a taxa de cesarianas no Brasil foi de 59,6% em 2023, sendo 49,5% no setor público e 69,5% nos serviços privados (alguns estabelecimentos de saúde particulares, a propósito, realizaram 100% de cesáreas). Trata-se de patamares muito acima da recomendação da OMS, que estabelece que apenas entre 10% e 15% dos partos devem ser cirúrgicos para garantir segurança materno-neonatal. As cesarianas desnecessárias aumentam os riscos de complicações para a mãe e o bebê, incluindo infecções, hemorragias e dificuldades respiratórias neonatais ^5^ ^,^ ^6^. Por outro lado, o modelo assistencial dos CPNp, ao incentivar o parto vaginal espontâneo, o respeito às escolhas da mulher e a promoção de um ambiente de cuidado humanizado, contribui diretamente para a redução dessas complicações, oferecendo um cuidado seguro e baseado em evidências científicas.

A implementação dos CPNp no Brasil está vinculada à Rede Cegonha, recentemente reformulada para Rede Alyne, como uma estratégia de reestruturação do modelo de atenção à saúde materna e infantil no Brasil. Esta rede temática e prioritária do Sistema Único de Saúde (SUS) visa garantir às mulheres o direito ao planejamento reprodutivo, à atenção humanizada durante a gravidez, ao parto e ao puerpério, e às crianças, o direito ao nascimento seguro e ao desenvolvimento saudável. Nesse sentido, os CPNp estão inseridos como um dos pilares dessa rede, com foco na promoção do parto humanizado, no respeito às escolhas da mulher e na redução das intervenções desnecessárias ^7^. 

Além das diretrizes nacionais, a atuação dos CPNp no Brasil tem consonância com as metas globais de saúde materna estabelecidas pela OMS. A organização defende que o parto em CPNp, conduzido por enfermeiras obstétricas e obstetrizes, tem o potencial de reduzir a morbimortalidade materna e neonatal em contextos de risco habitual ^8^. Estudos internacionais corroboram essa abordagem, demonstrando que partos realizados em unidades de cuidados de baixo risco apresentam desfechos igualmente seguros, ou até melhores, quando comparados aos realizados em ambientes hospitalares convencionais, desde que haja infraestrutura adequada e critérios rigorosos para a admissão das gestantes ^9^. A abordagem é reforçada pelas evidências que mostram que os partos assistidos por obstetrizes e enfermeiras obstétricas são associados a menores taxas de intervenções, como cesarianas, episiotomias e uso de ocitocina, além de promover maior satisfação materna e melhores desfechos neonatais ^10^ ^,^ ^11^.

O estudo *Nascer no Brasil II*, um amplo inquérito nacional, tem como um de seus principais objetivos avaliar a assistência ao pré-natal, parto, nascimento e ao pós-parto em hospitais públicos, mistos e privados no Brasil ^12^. Aninhado a essa pesquisa, o *Nascer nas Casas de Parto do Brasil* propõe analisar a estrutura, o modelo assistencial e os resultados da assistência ao pré-natal, parto e puerpério nos CPNp e compará-los com os dos hospitais públicos que participam do *Nascer no Brasil II*. Este estudo possibilitará uma compreensão detalhada do funcionamento dos CPNp e de seus resultados assistenciais, oferecendo subsídios para a avaliação desse modelo de cuidado no Brasil. A análise dos indicadores de monitoramento e avaliação dos CPNp, baseados nas diretrizes estabelecidas pela *Portaria nº 11/2015* ^2^, é fundamental para verificar se esses centros estão cumprindo seus objetivos de oferecer uma assistência de qualidade e segura às gestantes e recém-nascidos de risco habitual.

Diante desse cenário, o objetivo deste estudo é analisar os indicadores de monitoramento e avaliação dos CPNp participantes da pesquisa *Nascer nas Casas de Parto do Brasil*, tendo como referência a Portaria Ministerial para implantação e habilitação de CPNp no âmbito do SUS. 

## Métodos

### Tipo de estudo

O *Nascer nas Casas de Parto do Brasil* é um estudo transversal, descritivo e analítico, de abrangência nacional, alinhado ao *Nascer no Brasil II*. O protocolo com a metodologia do estudo está disponível em Leister et al. ^13^.

### Local do estudo

Fazem parte do *Nascer nas Casas de Parto* todos os oito CPNp do país que registraram, em 2019, mais de 100 partos no Sistema de Informações sobre Nascidos Vivos (SINASC) (Quadro 1). À época da seleção, havia 13 CPNp cadastrados no Cadastro Nacional de Estabelecimentos de Saúde (CNES), porém somente oito foram elegíveis para o estudo.

**Quadro 1:** Estabelecimentos participantes da pesquisa *Nascer nas Casas de Parto do Brasil*.

ESTABELECIMENTO	CIDADE/UF
CPNp1	São Paulo/São Paulo
CPNp2	Rio de Janeiro/Rio de Janeiro
CPNp3	Brasília/Distrito Federal
CPNp4	São Paulo/São Paulo
CPNp5	Paudalho/Pernambuco
CPNp6	Castanhal/Pará
CPNp7	Belo Horizonte/Minas Gerais
CPNp8	Salvador/Bahia

CPNp: Centros de Parto Normal Peri-hospitalares; UF: Unidade Federativa.

### Participantes do estudo

Oito gestoras participaram da pesquisa, cada uma representando um dos CPNp incluídos no *Nascer nas Casas de Parto*. As participantes foram identificadas quando da assinatura da carta de anuência para a participação no estudo. 

### Coleta dos dados

Foram coletados dados entre agosto de 2022 e março de 2023, por intermédio da plataforma eletrônica REDCap (*Research Electronic Data Capture*, https://redcapbrasil.com.br/), sendo o link de acesso ao instrumento disponibilizado às gestoras, que também participaram de entrevistas virtuais. Individualmente, elas foram convidadas a responder as perguntas sobre os indicadores de monitoramento e a avaliação dos CPNp e, também, sobre a caracterização e funcionamento destes, incluindo: serviços disponíveis, comissões e estrutura física; relacionamento com o usuário; equipamentos de assistência, incluindo seu uso diário e acessibilidade; medicamentos disponíveis; recursos humanos e capacitação; organização e processo de trabalho, incluindo protocolos assistenciais; critérios de admissão; equipamentos para atendimento às urgências e emergências; capacidade instalada de leitos; adaptações diante da pandemia de COVID-19.

Após preenchimento inicial e, de acordo com a necessidade de cada participante, foram realizadas reuniões com a equipe de pesquisa para esclarecer dúvidas relativas aos dados disponíveis e ao preenchimento no instrumento.

Neste artigo, vamos reportar os dados que se referem aos indicadores de monitoramento e a avaliação dos CPNp constantes no anexo II da *Portaria nº 11/2015* ^2^ que redefine as diretrizes para implantação e habilitação de CPNp no âmbito do SUS. Outros dados coletados serão publicados posteriormente e comparados com os resultados do *Nascer no Brasil II*.

### Variáveis do estudo

As variáveis correspondem a todos os 12 indicadores de monitoramento e avaliação dos CPNp ^2^ que serão divididos em:

#### Dados sobre os partos

Indicador 1: 

(1a) número de partos atendidos; 

(1b) percentual de partos atendidos por profissional de enfermagem e da medicina; 

(1c) percentual de partos de adolescentes (neste estudo foi considerado o percentual de adolescentes admitidas nos CPNp); 

(1d) percentual de partos na água;

(1e) percentual de partos em posição vertical.

#### Dados sobre o períneo

Indicador 2: percentual de episiotomia; 

Indicador 3: percentual de lacerações de 1º e 2º graus; 

Indicador 4: percentual de lacerações de 3º e 4º graus.

#### Dados sobre intervenções

Indicador 5: percentual de amniotomia/rotura artificial de membrana;

Indicador 6: percentual de parto com ocitocina no 2º estágio do parto.

#### Dados sobre acompanhante

Indicador 7: percentual de mulheres com acompanhante durante o trabalho de parto, parto e puerpério.

#### Dados sobre o acompanhamento pré-natal

Indicador 8: percentual de mulheres atendidas com seis ou mais consultas de pré-natal.

#### Dados sobre o recém-nascido:

Indicador 9:

(9a) percentual de recém-nascido com peso ao nascer < 2.500g e > 4.000g;

(9b) percentual de recém-nascido com idade gestacional < 37 semanas e > 41 semanas;

(9c) percentual de recém-nascido com Apgar < 7 no 5º minuto;

(9d) percentual de recém-nascido com contato pele a pele ininterrupto e imediato após o nascimento.

#### Dados sobre as transferências

Indicador 10: percentual de transferência intraparto, discriminado por motivo da transferência.

Indicador 11: 

(11a) percentual de transferência pós-parto, discriminado por motivos da transferência;

(11b) percentual de transferência neonatal, discriminado por motivos da transferência.

#### Dados sobre atendimento médico

Indicador 12: percentual de avaliação/procedimento médico obstétrico ou pediátrico no CPNp (refere-se à proporção de avaliações ou procedimentos realizados por médico obstetra ou pediatra nas dependências do próprio CPNp).

### Análise dos dados

Os dados foram analisados de forma descritiva, com valores absolutos e relativos e valores mínimo e máximo para cada indicador. 

### Aspectos éticos

O estudo seguiu todos os aspectos éticos da *Resolução CNS nº 466/2012* ^14^, e foi aprovado pelos Comitês de Ética em Pesquisa da Escola Nacional de Saúde Pública Sergio Arouca da Fundação Oswaldo Cruz (ENSP/Fiocruz, parecer nº 5.192.349); Fundação de Ensino e Pesquisa em Ciências da Saúde, Secretaria Estadual de Saúde do Distrito Federal (FEPECS/SES/DF, parecer nº 5.243.029); Hospital Sofia Feldman/Fundação de Assistência Integral à Saúde (parecer nº 5.256.617); Secretaria Municipal de Saúde do Rio de Janeiro (SMS/RJ, parecer nº 5.218.319); Secretaria Municipal da Saúde de São Paulo (SMS/SP, parecer nº 5.251.421). Ao serem convidadas para a pesquisa, as gestoras assinaram o Termo de Consentimento Livre e Esclarecido. Para este estudo, a pedido dos Comitês de Ética em Pesquisa, os dados dos CPNp serão apresentados e analisados agrupadamente.

## Resultados

Os dados foram coletados a partir do controle mais recente apresentado pelas gestões e correspondem a um período de 12 meses. Em sete CPNp, as informações referem-se ao ano de 2021 e em um deles, ao ano de 2020. 

Nos oito CPNp, o número de partos no período estudado foi de 3.097 (Indicador 1a). As admissões de gestantes se referem aos sete CPNp que informaram o dado, totalizando 3.367. De acordo com a Portaria Ministerial ^2^, há indicadores que devem ser calculados com base no número de partos, outros, porém, pelo número de admissões. Isto se deve porque algumas mulheres assistidas no CPNp podem ser transferidas para o hospital de referência antes do parto. Assim, o denominador (se pelo número de partos ou pelo número de admissões) estará indicado no rodapé das tabelas. Além disso, cabe esclarecer que, sendo os resultados apresentados agrupadamente, podem haver variações no número de admissões e de partos quando estes não se referirem a totalidade dos CPNp.

Na Tabela 1, estão descritos os partos de adolescentes (9,4%), partos atendidos por enfermeiras/obstetrizes (96,9%) e por médicos (3,1%), acompanhante de escolha da mulher (98,7%), gestantes que tiveram seis ou mais consultas pré-natais (74,6%) e aquelas que passaram por avaliação ou procedimento médico durante a internação no CPNp (0,2%). Para os indicadores “admissões de adolescentes” e “avaliação ou procedimento médico”, uma das instituições não têm esses dados coletados de forma rotineira, apesar de ter as informações disponíveis em prontuário.

**Tabela 1:** Indicadores gerais.

Indicador	N Denominador	n Indicador	%	IC95%
Partos (Indicador 1a)	3.097	-	-	-
Admissões de adolescentes (Indicador 1c) *	3.111	292	9,4	2,1-14,6
Partos por enfermeiras/obstetrizes (Indicador 1b)	3.097	3.001	96,9	83,9-100,0
Partos por médicos (Indicador 1b)	3.097	96	3,1	0,0-16,1
Acompanhante (Indicador 7)	3.097	3.058	98,7	90,9-100,0
≥ 6 consultas pré-natais (Indicador 8) *	3.111	2.320	74,6	46,5-96,6
Avaliação ou procedimento médico (Indicador 12) **	2.512	6	0,2	0,0-0,7

IC95%: intervalo de 95% de confiança; CPNp: Centros de Parto Normal Peri-hospitalares.

Nota: itens 1a, 1b, 1c, 7, 8 e 12 da *Portaria nº 11/2015* (Anexo II) ^2^.

* Dados analisados de acordo com as admissões em 6 CPNp;

** Dados analisados de acordo com o total de partos em 7 CPNp.

Quanto aos indicadores de assistência materna, como uso de amniotomia (15,7%), ocitocina (7%) e episiotomia (0,4%), bem como aos desfechos perineais (32,2% de períneo íntegro) e posições da mulher no parto (60,2% de parto verticalizado), estes encontram-se descritos na Tabela 2. Um dos CPNp não coleta de forma rotineira dados sobre lacerações simples (1º ou 2º graus) e nem sobre a posição do parto ou ocorrência em parto na água. Dois CPNp coletam de forma rotineira somente dados dos partos que ocorreram no CPNp, sem considerar as práticas após a transferência intraparto. Assim, dados em relação ao uso de ocitocina intraparto e realização de amniotomia estavam disponíveis em apenas seis CPNp

**Tabela 2:** Indicadores de assistência materna.

Indicador	N Denominador	n Indicador	%	IC95%
Amniotomia (Indicador 5) *	3.111	487	15,7	4,6-32,2
Ocitocina (Indicador 6) *	3.111	217	7,0	2,5-25,3
Partos na água (Indicador 1d) **	3.097	378	12,2	0,0-32,7
Partos verticais (Indicador 1e) ***	2.783	1.676	60,2	24,8-99,6
Períneo íntegro ***	2.783	896	32,2	14,9-46,6
Episiotomia (Indicador 2) **	3.097	11	0,4	0,0-3,1
Laceração 1º grau (Indicador 3) ***	2.783	1.234	44,3	25,6-59,9
Laceração 2º grau (Indicador 3) ***	2.783	608	21,8	11,3-50,6
Laceração 3º grau (Indicador 4) **	3.097	34	1,1	0,0-2,7
Laceração 4º grau (Indicador 4) **	3.097	2	0,1	0,0-0,2

IC95%: intervalo de 95% de confiança; CPNp: Centros de Parto Normal Peri-hospitalares.

Nota: itens 1d, 1e, 2, 3, 4, 5 e 6 da *Portaria nº 11/2015* (Anexo II) ^2^.

* Dados analisados de acordo com as admissões em 6 CPNp;

** Dados analisados de acordo com o total de partos em 8 CPNp;

*** Dados relativos ao número de partos em 7 CPNp.

Na Tabela 3, são apresentados os indicadores de assistência neonatal, como idade gestacional no parto (0,4% com < 37 semanas), Apgar no 5º minuto de vida (0,6% < 7) e contato pele a pele imediatamente ao nascer (97,6%). Um CPNp não registra rotineiramente idade gestacional fora do termo < 37 ou > 41 semanas. Por este motivo, para estes indicadores, foram considerados apenas sete CPNp.

**Tabela 3:** Indicadores neonatais.

Indicador	N Denominador	n Indicador	%	IC95%
Peso ao nascer < 2.500g (Indicador 9a) *	3.097	68	2,2	0,0-3,3
Peso ao nascer > 4.000g (Indicador 9a) *	3.097	67	2,2	0,8-4,8
RN com idade gestacional < 37 semanas (Indicador 9b) **	2.783	11	0,4	0,0-1,9
RN com idade gestacional > 41 semanas (Indicador 9b) **	2.783	53	1,9	0,0-8,5
Apgar < 7 no 5º minuto (Indicador 9c) *	3.097	18	0,6	0,0-1,3
Contato pele a pele imediato ao nascer (Indicador 9d) *	3.097	3.023	97,6	93,3-100

IC95%: intervalo de 95% de confiança; CPNp: Centros de Parto Normal Peri-hospitalares.

Nota: itens 9a, 9b, 9c, 9d da *Portaria nº 11/2015* (Anexo II) ^2^.

* Dados analisados de acordo com o total de partos dos 8 CPNp;

** Dados relativos ao número de partos em 7 CPNp.

Por fim, verificam-se os indicadores de transferências intraparto, pós-parto e neonatais e os respectivos motivos destas (Tabela 4), com destaque à solicitação materna por analgesia (4,6%) e desconforto respiratório do bebê (1,9%). Para as transferências intraparto, os indicadores foram calculados de acordo com o número de admissões; já para as transferências pós-parto e neonatal, o cálculo se deu pelo número de partos. Três CPNp não coletam rotineiramente os motivos de transferência. Por este motivo, os dados disponíveis variam de acordo com o tipo de transferência. Um dos CPNp não tinha disponível as taxas de transferências, somente dados dos partos ocorridos no local.

**Tabela 4:** Indicadores relativos às transferências intraparto, pós-parto e neonatais e seus respectivos motivos.

Transferência e motivo	N Denominador	n Indicador	%	IC95%
Transferência intraparto * (Indicador 10)	3.367	584	17,3	6,0-53,5
Motivos das transferências intraparto **	2.283			
Solicitação de analgesia		105	4,6	0,0-9,7
Parada de progressão		82	3,6	2,3-5,5
Alteração dos batimentos cardíacos fetais		76	3,3	0,4-7,5
Líquido meconial		42	1,8	0,0-2,7
Bolsa rota prolongada		25	1,1	0,0-3,9
Pico hipertensivo ou alterações de sinais vitais		18	0,8	0,0-2,1
Solicitação materna		5	0,2	0,0-0,9
Apresentação anômala		2	0,1	0,0-0,2
Sangramento durante o trabalho de parto		2	0,1	0,0-0,2
Outros		18	0,8	0,0-1,4
Transferência pós-parto *** (Indicador 11a)	2.783	71	2,6	1,2-4,2
Motivos das transferências pós-parto ^#^	2.264			
Hemorragia pós-parto		24	0,9	0,0-1,2
Pico hipertensivo ou alterações de sinais vitais		11	0,4	0,0-1,2
Retenção placentária		9	0,3	0,0-0,6
Suspeita de infecção		6	0,2	0,0-1,2
Laceração 3º ou 4º grau		5	0,2	0,0-0,5
Anemia por hemorragia pós-parto		4	0,1	0,0-0,7
Hematoma perineal		2	0,1	0,0-0,2
Laceração cervical		2	0,1	0,0-0,2
Outros ^##^		3	0,1	0,0-0,5
Transferência neonatal ^#^ (Indicador 11b)	2.664	115	4,3	1,9-8,3
Motivos das transferências neonatais ^#^	2.664			
Desconforto respiratório		50	1,9	1,1-3,1
Icterícia precoce		20	0,8	0,0-1,4
Suspeita ou confirmação de infecção		16	0,6	0,0-1,2
Alterações cardíacas		8	0,3	0,0-0,9
Baixo peso		7	0,3	0,0-0,9
Hipoglicemia		5	0,2	0,0-0,5
Suspeita de alterações congênita		4	0,2	0,0-0,5
Outros ^###^		5	0,2	0,0-0,6

IC95%: intervalo de 95% de confiança; CPNp: Centros de Parto Normal Peri-hospitalares.

Nota: itens 10, 11a e 11b da *Portaria nº 11/2015* (Anexo II) ^2^.

* Dados relativos às admissões em 7 CPNp;

** Dados relativos às admissões em 4 CPNp;

*** Dados relativos aos partos ocorridos em 7 CPNp;

^#^ Dados relativos aos partos ocorridos em 6 CPNp;

^##^ Necessidade de avaliação de laceração perineal complexa de 2º grau, falta de energia elétrica no CPNp, paciente de alto risco transferida após admissão em expulsivo;

^###^ Febre, hipotermia, necessidade de reanimação, fratura de clavícula e reação vacinal.

Embora não previstos na *Portaria nº 11/2015* ^2^, dois indicadores igualmente relevantes foram coletados nos CPNp participantes do estudo: a mortalidade materna e a mortalidade neonatal. No período analisado, não houve mortes de mulheres assistidas nos CPNp, mas ocorreram uma morte fetal durante o trabalho de parto (natimorto) e uma morte neonatal, em CPNp distintos, o que corresponde a 0,6% dos nascimentos ocorridos nas instituições. 

## Discussão

Estabelecida em suas diretrizes de implementação ^2^, a estrutura física dos CPNp possui capacidade para atender de 40 a 70 partos mensais, a depender do número de quartos PPP (pré-parto, parto e pós-parto) de cada unidade. Neste estudo, identificou-se relativa subutilização destes serviços, haja vista o quantitativo de partos registrados no período de um ano (N = 3.097; média de 32 partos mensais por instituição), o que representa apenas 48,5% da capacidade mensal média de partos dos oito CPNp e apenas 0,1% do total de nascimentos em território brasileiro. Estes números são o reflexo da cultura hospitalocêntrica, medicalizada e médico-centrada predominante no país ^1^ ^,^ ^15^ que retira das parturientes e bebês saudáveis a possibilidade de vivenciarem seus partos e nascimentos em um CPNp, instituição cujo ambiente e assistência são seguros e apoiados pelas melhores evidências científicas ^16^ ^,^ ^17^ ^,^ ^18^ ^,^ ^19^.

Outro fator que responde por este cenário é a supervalorização das cesarianas. Há anos o Brasil apresenta índices inaceitáveis de cesáreas, chegando a quase 70% no setor privado e 50% no público ^1^ ^,^ ^4^ ^,^ ^20^, ignorando os alertas da OMS e da comunidade científica sobre a ausência de benefícios e maiores riscos maternos e neonatais quando a taxa deste procedimento supera os 15% ^5^ ^,^ ^21^ ^,^ ^22^.

Desde que foram regulamentados, os CPNp podem prescindir da figura do médico na assistência, ou seja, a equipe mínima para implementação e manutenção do serviço é composta por obstetriz/enfermeira obstétrica, técnico de enfermagem e auxiliar de serviços gerais ^2^. Isto explica a razão pela qual os resultados deste estudo mostram que 96,9% dos partos foram conduzidos por obstetriz ou enfermeira obstétrica. 

Os CPNp caracterizam-se pela atenção às gestantes, parturientes, puérperas e bebês de risco habitual ^23^. O cuidado proporcionado por essas/es profissionais é associado a desfechos maternos e neonatais positivos. Um estudo sobre os resultados da assistência em diferentes modelos de atenção identificou que o modelo liderado por obstetrizes e enfermeiras obstétricas está associado a maiores chances de parto vaginal espontâneo e satisfação materna, bem como de menor taxa de partos instrumentais, redução de nascimentos pré-termos e de perdas e mortes fetais e neonatais ^24^.

Os CPNp possuem critérios bem estabelecidos para admissão de gestantes e parturientes, garantindo assistência exclusiva ao risco habitual. No Município de São Paulo, foi publicado o *Manual Técnico das Casas de Parto* ^25^, que contém a descrição dos critérios de elegibilidade para admissão nos serviços. Um deles é a idade da gestante, que deve ser ≥ 15 anos, já que gestações nos extremos da idade reprodutiva (< 15 e > 40 anos) são classificadas como de alto risco ^26^. No presente estudo, apenas 9,4% dos partos foram de adolescentes, um percentual relativamente menor do que aqueles observados na média nacional (12,3% em 2022) ^19^. Esta diferença pode indicar que os CPNp seguem os critérios de elegibilidade pré-estabelecidos.

Um importante indicador observado neste estudo foi a presença de um acompanhante durante o trabalho de parto, parto e pós-parto, tal como previsto em Lei Federal ^27^. No presente estudo, os CPNp garantiram este direito a todas as mulheres, registrando quase 99% de parturientes e puérperas acompanhadas. A relevância deste dado se deve ao fato de instituições hospitalares ainda restringirem ou até impedirem a presença de acompanhantes, a despeito dos avanços assistenciais dos últimos anos observados por Leal et al. ^28^. Segundo os autores, entre os anos de 2011 e 2017, a presença de acompanhante durante o parto vaginal aumentou em 164% no serviço público e 73% no setor privado, mas ainda carece de melhorias. Cabe ressaltar que a presença de acompanhante é potencialmente protetora das mulheres, seja evitando a violência institucional/obstétrica, seja proporcionando conforto e melhor satisfação materna ^29^.

Sobre o indicador do número de consultas pré-natais, sabe-se da relação direta entre desfechos maternos, fetais e neonatais positivos e um pré-natal de qualidade. Assim, recomenda-se pelo menos seis consultas durante a gestação para que doenças e intercorrências importantes possam ser rastreadas e identificadas a tempo de receberem tratamento ou intervenção oportuna, aumentando as chances de sobrevida materna e perinatal ^30^. Neste estudo, 74,6% de mulheres tiveram seis ou mais consultas pré-natais. Todavia, chama atenção a diferença entre a porcentagem mínima e máxima entre os CPNp (46,5% e 96,6%), convergindo com a realidade nacional díspar em relação ao acesso, cobertura e qualidade do pré-natal oferecidos às gestantes brasileiras ^31^.

Com relação aos indicadores de assistência materna, as intervenções avaliadas foram a amniotomia, a administração de ocitocina sintética durante o trabalho de parto e a episiotomia. Os três procedimentos, quando realizados de forma rotineira e/ou precocemente, são considerados potencialmente causadores de danos ^3^. 

O uso de amniotomia e ocitocina intraparto (média de 15,7% e 7%, respectivamente) é significativamente menor em CPNp do que em instituições hospitalares (40,7% e 38,2%, respectivamente) ^32^. Sobre a episiotomia, esta foi realizada em apenas 0,4% da amostra deste estudo, contrastando com a taxa de 56% encontrada nos hospitais investigados no *Nascer no Brasil I* ^32^ e apesar dos avanços na redução deste procedimento, nos últimos anos, observados por Leal et al. ^28^. Esta discrepância revela que os CPNp estão alinhados às sólidas evidências que atestam e ratificam a inexistência de benefícios relacionados à prática da episiotomia ^3^ ^,^ ^33^, e que o modelo biomédico hospitalar continua violando o direito à integridade corporal das mulheres ^34^. 

Outro dado relevante dos indicadores de assistência materna são as lacerações perineais. Do total de mulheres assistidas nos CPNp, 76,5% delas não precisaram de sutura, sendo 32,2% de períneo íntegro e 44,3% de lacerações de 1º grau. Além disso, um percentual pequeno de parturientes sofreram lacerações graves, de 3º e 4º graus (1,1% e 0,06%, respectivamente). Estes dados vão ao encontro de estudo sobre a implementação de um protocolo de episiotomia zero, onde foi observado que a maior parte das mulheres teve períneo íntegro ou laceração de 1º grau ^35^. 

Com relação à posição materna, os CPNp registraram 60,2% de partos verticalizados. As posições não litotômicas são fortemente recomendadas pela OMS durante o trabalho de parto e parto. Além disso, para uma experiência positiva, a OMS sugere que seja respeitada a escolha da mulher quanto à posição que lhe traga mais conforto ^3^. Entretanto, estudo com mais de 3 mil parturientes assistidas em 606 hospitais públicos e privados em todo o país identificou apenas 6,7% de partos verticais ^36^. 

Quanto aos indicadores neonatais dos CPNp, é esperado que nasçam poucos bebês pré ou pós-termo, bem como pequenos ou grandes para a idade gestacional, tal como ocorreu neste estudo. Isto ocorre porque os critérios de admissão das parturientes nessas instituições ^25^ são rigorosos, a exemplo de não poderem dar à luz nestes locais mulheres com idade gestacional inferior a 37 semanas, bem como mulheres cujos resultados dos exames pré-natais estão alterados.

Apenas 0,6% (n = 18) dos bebês que nasceram nos CPNp deste estudo apresentaram Apgar < 7 no 5º minuto de vida. A despeito da subjetividade na composição do Apgar, ele ainda é o escore universalmente utilizado para avaliação da condição do bebê ao nascer ^37^. Estudos internacionais realizados com população de baixo risco ^38^ ^,^ ^39^ não encontraram diferenças entre a prevalência do Apgar < 7 no 5º minuto de bebês nascidos em CPNp ou hospitais. 

O último indicador neonatal de avaliação dos CPNp é o contato pele a pele entre mãe e recém-nascido imediatamente após o parto. Trata-se de um indicador de grande significado para avaliação das instituições de saúde, haja vista que os benefícios desta intervenção podem abarcar desde o equilíbrio termodinâmico do recém-nascido, até a oportunidade de estímulo à amamentação e à criação do vínculo entre mãe e bebê ^3^ ^,^ ^40^. Neste estudo, 97,6% dos bebês foram colocados em contato pele a pele com suas mães após o nascimento - percentual consideravelmente maior do que o encontrado por estudo ^41^ que avaliou práticas realizadas com recém-nascidos saudáveis em instituições hospitalares do país (contato pele a pele em apenas 28,2% dos nascimentos). 

O último bloco de avaliação dos indicadores dos CPNp se refere às transferências maternas e neonatais. Em um cenário cujo trabalho de parto/parto/pós-parto/nascimento está saindo dos padrões de normalidade, o encaminhamento da mãe e/ou do bebê deve ser feito para o hospital de referência em segurança e oportunamente. Por esta razão, justifica-se que a instalação dessas instituições ocorra nas imediações de um serviço de maior densidade tecnológica, para que haja continuidade do cuidado e resguardo da saúde e do bem-estar materno-fetal/neonatal. Neste estudo, as transferências intraparto foram maiores que as transferências pós-parto e neonatais (17,3%, 2,6% e 4,3% respectivamente). Estes dados corroboram outras pesquisas que avaliaram esse indicador, como a de Mendonça et al. ^42^, que identificaram 25,6% de transferências intraparto e 1,4% no pós-parto, e o estudo de Pereira et al. ^43^, cujas transferências neonatais representaram 6,6% dos nascimentos em uma Casa de Parto de Minas Gerais. 

Dos motivos de transferências intraparto, destacou-se a solicitação materna por analgesia (n = 105, 4,6%). Este achado corrobora a experiência da Austrália e Inglaterra, em que houve, respectivamente, 6,1% e 6,5% de transferências maternas por solicitação de epidural ^44^ ^,^ ^45^. Ainda que expressos por uma dimensão subjetiva, são inquestionáveis a dor e o cansaço das mulheres em trabalho de parto ‒ condições que são minimizadas com o bloqueio temporário da dor. Enquanto intervenção de maior complexidade, a analgesia não faz parte do rol de procedimentos dos CPNp, sobretudo porque estes se caracterizam como um modelo de atenção que dispensa a presença de médicos, que são os profissionais habilitados para esta intervenção ^2^. Cabe ressaltar, no entanto, que as Casas de Parto oferecem diferentes métodos não farmacológicos para alívio das dores, como banho de imersão e massagem, que podem contribuir para o baixo percentual de mulheres que optam pela transferência para o hospital para receberem analgesia.

Quanto às razões para transferências pós-parto e neonatais, a hemorragia materna (n = 24, 0,9%) e o desconforto respiratório neonatal (n = 50, 1,9%) foram os mais frequentes. Estudo sobre transferências de um CPNp identificou taxa de 0,3% por motivo de hemorragias maternas e 4,3% por desconforto respiratório neonatal ^42^. Ressalta-se que as transferências por hemorragia pós-parto acontecem nas Casas de Parto para aquelas com alteração de sinais vitais e/ou hemodinâmicas, ou sem essas alterações, mas com Hb ≤ 7g/dl. Além disso, todos os bebês nascidos em CPNp são de termo. No cenário hospitalar, espera-se que essas intercorrências sejam mais frequentes, uma vez que o perfil de atendimento contempla o alto risco. Betti et al. ^46^, por exemplo, identificaram 23,5% de hemorragia pós-parto em mulheres que vivenciaram o parto normal em um hospital universitário. Bernardino et al. ^47^ encontraram 12,1% de desconforto respiratório em bebês a termo nascidos em hospital de Vilarejo de Cuiabá (Gouveia, Minas Gerais). 

Por fim, convém examinar a mortalidade nos CPNp estudados (nenhuma morte materna e 0,6% de mortalidade neonatal). Evidentemente, espera-se maior frequência de mortes nos hospitais ^48^ ^,^ ^49^, frente à relação direta do risco obstétrico com este evento. Entretanto, o excesso de intervenções no parto de mulheres de risco habitual, como cesarianas desnecessárias e uso indiscriminado de ocitocina, também podem acarretar complicações a mães e bebês saudáveis ^32^. Assim, diante do risco habitual, um modelo de atenção que privilegia o uso criterioso e alinhado às evidências científicas das intervenções, tal como os CPNp, é potencialmente protetor do bem-estar e da vida das mães e bebês.

Alguns dos indicadores de monitoramento e avaliação previstos pela portaria que habilita os CPNp não foram informados por todas as instituições participantes deste estudo, tal como as admissões de adolescentes, algumas razões das transferências para o hospital de referência e o uso de ocitocina e amniotomia. Este dado reflete tanto uma limitação do estudo quanto a dificuldade e a necessidade de padronização dos processos de gestão. A maioria dos dados faltantes, relativos às transferências e as práticas de continuidade da assistência na rede de referência, indica uma possível dificuldade de comunicação e trabalho colaborativo entre o CPNp e o hospital, que deve ser melhor investigada em estudos futuros.

A indisponibilidade dessas informações, previstas em portaria ministerial e diretriz nacional, reflete diretamente na avaliação e monitoramento da segurança e da qualidade assistencial, bem como na transparência dos dados para a sociedade e para a formulação e manutenção das políticas de incentivo ao modelo. Uma vez habilitados antes da *Portaria nº 11/2015* ^2^, nem todos os CPNp pertenciam à Rede Cegonha. Possivelmente, este é o motivo pelo qual alguns indicadores não estavam sendo coletados rotineiramente por parte das instituições. Cabe relatar, porém, que durante as entrevistas com as gestoras, três delas reconheceram a necessidade de revisão dos processos de gestão relativos ao registro e controle dos indicadores de monitoramento dos CPNp. 

## Conclusão

Este estudo, pioneiro em analisar nacionalmente os indicadores assistenciais dos CPNp, identificou que, embora haja relativa subutilização destes estabelecimentos de saúde no país, eles oferecem às mães e bebês de risco habitual uma assistência segura, respeitosa, humano-centrada e baseada nas melhores evidências científicas, ratificadas pela OMS e pelo Ministério da Saúde brasileiro. 

Apesar dos resultados positivos observados neste estudo, nota-se importante variação, entre os CPNp, em alguns dos indicadores, como amniotomia, uso de ocitocina e percentuais de partos na água e partos verticalizados. A inexistência de um manual técnico nacional das Casas de Parto, padronizando desde os critérios de admissão até os procedimentos realizados, pode responder por essas variações. Ademais, é relevante considerar as diferenças culturais no país, o que pode influenciar as preferências maternas e o modelo de formação dos profissionais atuantes nas instituições.

Recentemente, com a reformulação da rede prioritária do SUS para atenção materna e infantil (Rede Alyne ^7^), houve ênfase na urgência de implementação de um novo modelo de cuidado obstétrico e perinatal para a redução da mortalidade materna no país (especialmente da população negra) e para o alcance dos Objetivos de Desenvolvimento Sustentável (ODS). Para isto, uma das estratégias previstas na Rede Alyne foi o incremento de recursos, via Programa de Aceleração do Crescimento (PAC) do Governo Federal, para a construção de 30 novos CPNp em território nacional até 2026. Isto reforça a imprescindibilidade do modelo de atenção dos centros para o cuidado humanizado e de qualidade às mães e bebês, ratificada pelos resultados deste estudo.
